# Anti-Thyroid Peroxidase Reactivity Is Heightened in Pemphigus Vulgaris and Is Driven by Human Leukocyte Antigen Status and the Absence of Desmoglein Reactivity

**DOI:** 10.3389/fimmu.2018.00625

**Published:** 2018-04-05

**Authors:** Kristina Seiffert-Sinha, Shahzaib Khan, Kristopher Attwood, John A. Gerlach, Animesh A. Sinha

**Affiliations:** ^1^Department of Dermatology, Jacobs School of Medicine and Biomedical Sciences, Buffalo, NY, United States; ^2^Department of Biostatistics and Bioinformatics, Roswell Park Cancer Institute, Buffalo, NY, United States; ^3^Biomedical Laboratory Diagnostics Program, Tissue Typing Laboratory, Michigan State University, East Lansing, MI, United States

**Keywords:** pemphigus vulgaris, anti-thyroid peroxidase antibody, anti-thyroglobulin antibody, anti-desmoglein antibody, human leukocyte antigen

## Abstract

Pemphigus vulgaris (PV) belongs to an autoimmune disease cluster that includes autoimmune thyroid disease (AITD), suggesting common mechanisms driving autoimmune susceptibility. Our group has shown that PV patients exhibit significant reactivity to AITD-related anti-thyroid peroxidase (anti-TPO), and anti-TPO antibodies affect signaling pathways in keratinocytes similar to anti-desmoglein (Dsg) 3 antibodies. To further assess the relevance of anti-TPO reactivity in PV, we analyzed anti-TPO levels in 280 PV and 167 healthy control serum samples across a comprehensive set of variable and static parameters of disease activity and etiopathogenesis. PV patients have significantly higher activity rates (A.R.s) for anti-TPO than healthy controls, but levels do not differ between phases of clinical activity and remission. Patients that carry both the PV-associated human leukocyte antigen (HLA) alleles DRB1*0402 and DQB1*0503, or DQB1*0503 alone show a low prevalence of anti-TPO (A.R. 9.5 and 4.8%, respectively), while patients that lack expression of these alleles or carry DRB1*0402 alone have a much higher prevalence of anti-TPO (A.R. 23.1 and 15.8%, respectively), suggesting that the absence of DQB1*0503 may predispose patients to the development of anti-TPO antibodies. Similarly, anti-Dsg1^−^/3^−^ patients have a higher anti-TPO A.R. (26.9%) than anti-Dsg1^−^/3^+^ (18.8%), anti-Dsg1^+^/3^−^ (14.3%), and anti-Dsg1^+^/3^+^ (3.9%) patients. Our data suggest that anti-TPO reactivity in PV is driven by genetic markers that may be in linkage disequilibrium with the established PV-susceptibility alleles and that this association drives the selection of a combination of anti-Dsg and anti-TPO antibodies, with anti-TPO filling the gap in active patients that do not carry the established PV-associated autoantibodies and/or are lacking the established PV-HLA-susceptibility alleles.

## Introduction

It is well accepted that numerous autoimmune diseases can co-exist within individuals or certain families, a concept known as *autoimmune diathesis* (a broad genetic predisposition to develop autoimmune disease) (1, 2). Previous work from our lab and others has suggested that this is also the case for pemphigus vulgaris (PV), a devastating autoimmune bullous skin disorder characterized by intraepidermal acantholysis and blister formation in skin and mucous membranes (3–10). Among the autoimmune diseases found in PV patients and/or their family members, autoimmune thyroid disease (AITD) is the most common, followed by rheumatoid arthritis (RA) and diabetes mellitus type I (4, 10, 11). These data indicate that PV belongs to an established autoimmune disease cluster comprised of AITD, RA and type I diabetes, suggesting the possibility of common genetic elements across clinically distinct diseases that might underlie autoimmune susceptibility (4, 8). Interestingly, a co-occurrence of autoantibodies associated with PV, AITD and RA has also been described in a large sampling of healthy control blood exhibiting ANA positivity with lupus erythematosus-associated staining patterns, further indicating a shared control of production of these autoantibodies (12).

Susceptibility to disease is complex, including (mostly unknown) genetic and environmental factors. Numerous studies have established a strong association between specific human leukocyte antigen (HLA) class II alleles, namely, DRB1*0402 and DQB1*0503, and increased risk for PV (13–15). It has been postulated that the specific binding pockets formed by these HLA molecules direct the preferential presentation of certain self-peptides and in turn inform production of specific autoantibodies (16). However, the broader impact of PV-associated HLA alleles in the development of the spectrum of PV-associated autoantibodies is not known.

Historically, PV has been linked to autoantibodies primarily targeting the desmosomal adhesion molecules desmoglein (Dsg) 3 and, in some cases, Dsg1, two members of the superfamily of cadherin molecules integral to intracellular adhesive junctions (17–19), where they act by steric hindrance and/or induction of intracellular signaling mechanisms (20). However, a growing body of literature suggests reactivities in PV against additional, non-desmoglein autoantigens, among them thyroid peroxidase (TPO) and muscarinic acetylcholine receptors (21, 22). Ongoing research in our lab revealed that PV patients exhibit significant reactivity to TPO (22), and that anti-thyroid peroxidase (anti-TPO) antibodies can induce keratinocyte dissociation *in vitro* and affect signaling pathways in keratinocytes similar to those seen after binding of anti-Dsg3 antibodies (Sajda et al., manuscript in preparation). This body of work clearly warrants further investigation into the role of thyroid-related autoantibodies in the PV patient population.

Although it has been reported that the AITD-related autoantibodies anti-TPO and anti-thyroglobulin (anti-Tg) are more prevalent in PV patients than the general population (3, 5, 6, 9, 23), thus far, levels of anti-thyroid antibodies have not been associated with static variables such as HLA status and sex or with dynamic clinical parameters including disease activity, morphology, and anti-desmoglein reactivity. Moreover, the link between specific HLA alleles and anti-thyroid autoantibody profiles in PV patients has not been investigated.

In this study, we aimed to address these gaps in knowledge as well as validate the findings in previous studies in a larger and ethnically different patient population. For this purpose, we measured anti-TPO and anti-Tg antibody levels in 280 serum samples from 225 North American PV patients and 167 serum samples from 148 healthy controls, and analyzed them across a comprehensive set of variable and static parameters of PV disease activity and etiopathogenesis.

We confirm in our North American study population that anti-thyroid antibodies are more prevalent in PV patients as compared with healthy controls. Furthermore, we find significant associations between anti-thyroid autoantibody reactivity, HLA status and anti-Dsg antibody profiles, thus providing insight into the complex interplay between HLA, autoantibody selection, and clinical outcomes.

## Materials and Methods

### Patient Population

Two hundred twenty-five patients with a diagnosis of PV were enrolled in our database over a time span of more than 10 years from the dermatology outpatient clinics of Weill Medical College of Cornell University, Michigan State University and the University at Buffalo as well as annual meetings of International Pemphigus and Pemphigoid Foundation. The study was approved by the institutional review boards at Weill Cornell Medical College (IRB 0998-398), Michigan State University (IRB 05-1034), and the University at Buffalo (IRB 456887). The diagnosis of Pemphigus was based on clinical, histological, and/or serological criteria. All patients had a biopsy confirmed diagnosis of PV before enrollment in the study. Venous blood was drawn after obtaining written informed consent, and serum was separated and immediately stored at −80°C in our biorepository. Demographic information as well as information regarding disease activity, morphology, any reported comorbidities and family history was collected by a trained medical professional at the time of blood draw. Blood samples from 148 controls (both related and unrelated to pemphigus patients) were obtained as described earlier. Some of the patients and controls donated blood repeatedly. The maximum number of blood samples used in this study by any patient or control is 2, and the average number of samples per patients or controls is 1.14 and 1.13, respectively. The demographic information is summarized in Table 1.

**Table 1:** Study population demographic data and human leukocyte antigen (HLA) association.

	PV	CR
# of Patients	225	148
# of Samples	280	167
Ethnicity		
% African American	3.6	9.5
% Hispanic	9.7	3.4
% Asian	9.7	18.4
% Caucasian (non-Jewish)	42.5	43.5
% Ashkenazi (Jewish)	30.1	20.4
% Other	4.4	4.8
Age at blood draw (years ± SD)	52.9 ± 14.4	46.0 ± 18.7
Age at onset (years ± SD)	45.1 ± 13.9	n/a
Male:female ratio (*n*)	1:1.92 (M = 77; F = 148)	1:1.55 (M = 90; F = 58)
% Active (*n*)	47.5 (133)	n/a
% Remittent (*n*)	36.07 (101)	n/a
% HLA^+^ (*n*)	82.22 (185)	24.68
% HLA^−^ (*n*)	14.66 (33)	75.32

PV, pemphigus vulgaris; CR, healthy control; HLA^+^, carriers of the PV-associated HLA-susceptibility alleles DRB1*0402 and/or DQB1*0503; HLA^−^, carriers of any HLA allele except the PV-associated HLA-susceptibility alleles DRB1*0402 and/or DQB1*0503.

*Disease activity is determined as number of samples from patients in the active or remittent phase of disease out of 280 total samples (note: samples from patients with transient lesions only were not included in these analyses)*.

*HLA association was determined as number of patients carrying or lacking PV-associated HLA alleles out of the total number of 250 samples (note: DNA for HLA typing was not available in 7 cases)*.

### Disease Activity

Phases of disease activity were defined by consensus guidelines (24). Briefly, patients were considered to be in the *active* phase of disease if three or more non-transient lesions were present for more than a week. Patients with absence of new or established lesions for more than 4 weeks were considered *remittent* while patients with transient lesions (lasting less than a week) were deemed *partially remittent* (25). For the analyses involving disease activity, *partially remittent* patients were excluded from the analysis, as they did not fit either of the two main activity categories.

### Anti-TPO and Anti-Tg Enzyme-Linked Immunosorbent Assay (ELISA)

An ELISA for anti-TPO and anti-Tg antibodies was performed employing ELISA kits by GenWay Biotech (GWB-521202 and GWB-521201, respectively) using 1:101 serum dilution as per the manufacturer’s recommendations. Antibody positivity was defined as ELISA levels >35 AU/mL for anti-TPO antibodies and >10 AU/mL for anti-Tg antibodies.

### Anti-Dsg3 and Anti-Dsg1 ELISA

The anti-Dsg ELISA was performed employing Dsg1 and Dsg3 ELISA kits (MBL Intl., RG-7593D) as per the manufacturer’s guidelines using a 1:101 serum dilution. Serum samples with an anti-Dsg3 or anti-Dsg1 concentration of >150 U/ml were further diluted to avoid misinterpretation at the upper level of detectability for the ELISA kit. Antibody positivity for anti-Dsg1 and anti-Dsg3 was defined as ELISA levels >20 U/mL.

### HLA Typing

High resolution HLA typing was performed by PCR amplification with sequence-specific primers at the Histocompatibility and Immunogenetics Laboratory at Michigan State University (26, 27) using commercially available kits (One lambda, Thermo Fisher Scientific). Patients with one or both of the PV-associated HLA alleles DRB1*0402 and DQB1*0503 were labeled as HLA-positive while those without either of these two alleles were labeled as HLA-negative.

### Statistical Analysis

Patient demographics were reported as mean and SD for continuous data, and as frequencies and relative frequencies for categorical data.

The anti-TPO and anti-Tg antibody rates were modeled as a function of group (disease status, gender, HLA type, and anti-Dsg1/3 status) using a generalized estimating equations (GEE) logistic regression model. The GEE model takes into account the repeated measures collected on some patients. Estimates of the activity rates (A.R.s) and corresponding 95% confidence intervals are obtained from the fitted models. A.R.s represent the estimated percent positive in a given population based on multiple observations (i.e., taking into consideration that some patients were sampled more than once). The association between HLA and Dsg status was evaluated using Fisher’s exact test. In a limited number of subjects, either DNA or serum was not available to perform all HLA typing and antibody determination, thus, the analyses listed reflect the number of patients in a given comparison where the required information was simultaneously accessible.

A descriptive cluster analysis of anti-TPO and anti-Tg antibody expression, Dsg1/3 status, and HLA type was performed using standard principle component methods. The overall variability explained by the optimal clustering model and the inter-cluster *R*^2^ for each variable are obtained. Factor scores were obtained using the standardized scoring coefficients associated with the optimal clustering model. The clusters are displayed graphically using a tree diagram and using a scatter plot of the corresponding factor scores.

All analyses were conducted in SAS v9.4 (Cary, NC, USA) at a significance level of 0.05.

## Results

### Correlation Between Reported History of AITD and Anti-Thyroid Antibody Profiles of PV Patients

**Figure 1:**
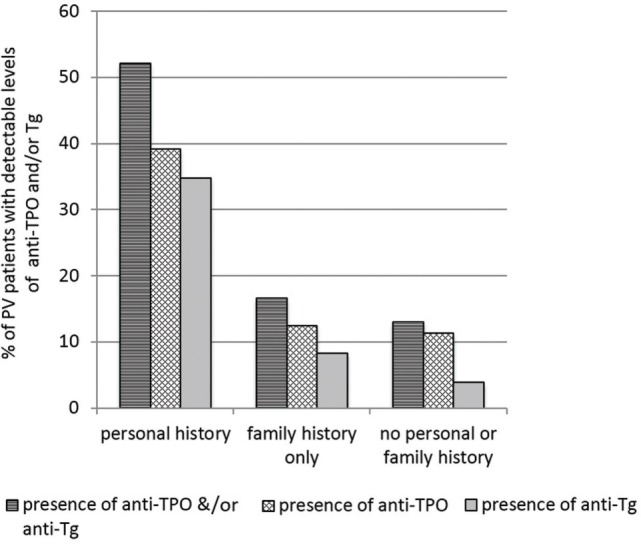
Levels of anti-thyroid peroxidase (anti-TPO) and or anti-thyroglobulin (anti-Tg) by self-reported presence of personal or family history of autoimmune thyroid disease (AITD) in pemphigus vulgaris (PV) patients. Bars represent the percentage of PV patients that have detectable levels of anti-TPO or anti-Tg in subgroups divided by personal and family history of AITD.

We previously examined the self-reported co-existence of other autoimmune conditions in PV patients and found AITD to be the leading comorbidity in three independent studies (4, 8, 10). In this study, out of a total of 225 PV patients enrolled, 10.2% (*n* = 23) reported a personal history of AITD and 15.55% (*n* = 35) reported a history of thyroid disease in family members regardless of personal history of AITD. Among those with a self-reported positive personal history of AITD, 52.17% (*n* = 12) had detectable anti-thyroid autoantibodies (either anti-TPO or anti-Tg) in their sera, with nearly equal numbers of patients carrying either anti-TPO antibodies or anti-Tg antibodies (Figure 1). Of those who reported only a family history of thyroid disease (i.e., no personal history) (*n* = 24), only 16.66% (*n* = 4) had anti-TPO or anti-Tg antibodies in their sera (two patients carried anti-TPO antibodies only, one patient carried anti-Tg Abs only, and one carried both anti-TPO and anti-Tg antibodies). Among PV patients that did *not* report a history of thyroid disease (*n* = 177), 12.99% (*n* = 23) were found to still carry autoantibodies directed against either/or anti-TPO and anti-Tg, albeit with considerably lower levels of anti-Tg (3.95%, *n* = 7). These data indicate that while PV patients with a history of thyroid-related symptoms are substantially more likely to carry autoimmune thyroid-related autoantibodies than PV patients with no thyroid-related symptoms, this correlation is not strong. Conversely, a proportion of PV patients with no personal- or family history of AITD still carries AITD-related autoantibodies at levels similar to those observed in PV patients with a family history of AITD.

### Anti-Thyroid Antibodies Are Related to Disease Expression, but Not Disease Activity

**Figure 2:**
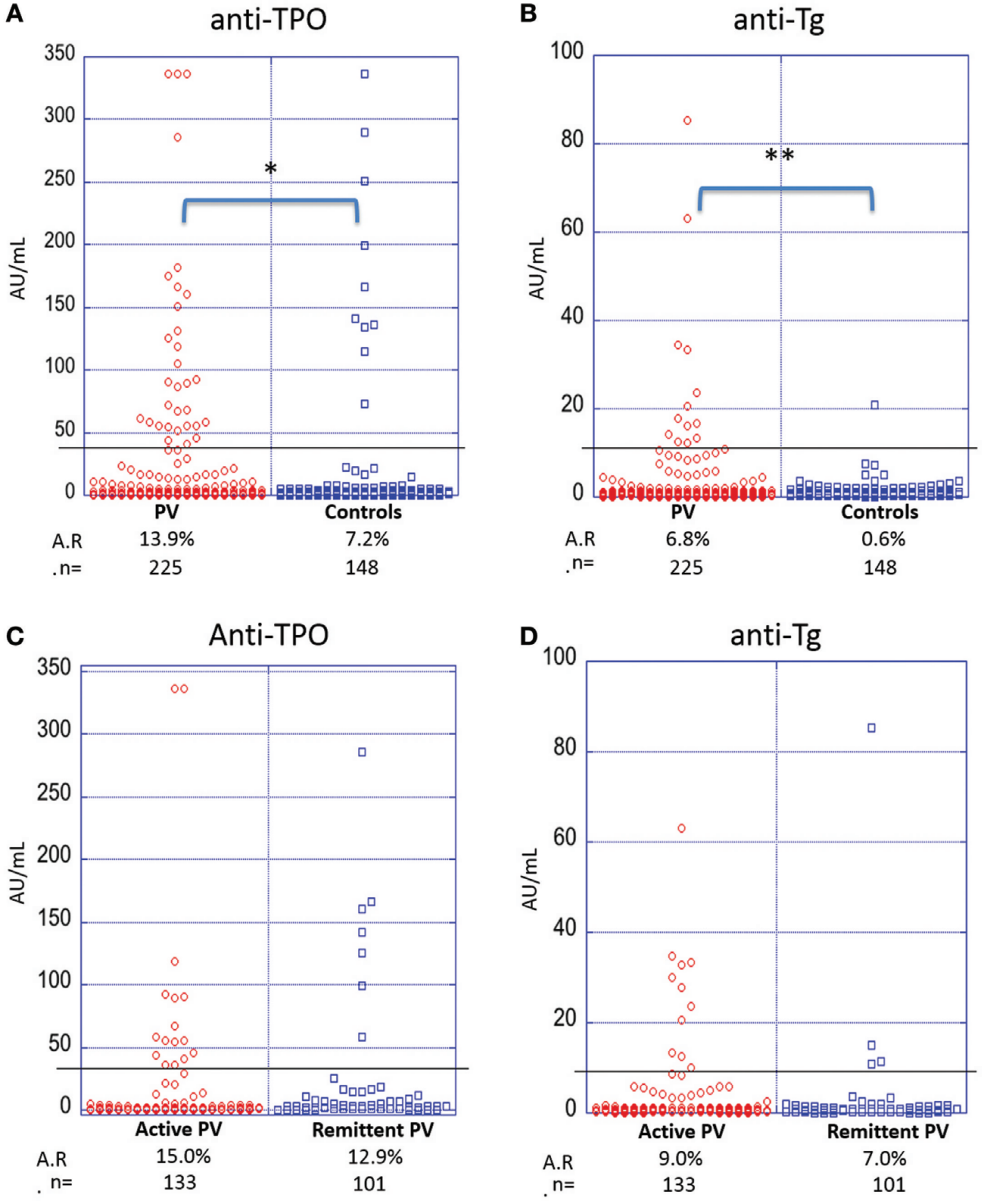
Activity rates of anti-thyroid peroxidase (anti-TPO) and anti-thyroglobulin (anti-Tg) based on disease state and disease activity. Individual dots represent anti-TPO and anti-Tg levels (in AU/ml) in pemphigus vulgaris (PV) patients vs. controls **(A,B)** and active vs. remittent PV patients **(C,D)** for anti-TPO **(A,C)** and anti-Tg **(B,D)** antibodies (*p < 0.05 and **p < 0.01). Abbreviation: A.R., activity rate. Anti-TPO and anti-Tg antibody rates were modeled as a function of disease status and activity using a generalized estimating equations logistic regression model.

To establish baseline and PV-related A.R.s in our study population, we determined levels of anti-TPO and anti-Tg reactivity in 225 PV patients compared with 148 healthy controls. We find that PV patients have a significantly higher prevalence of anti-TPO antibodies (A.R. 13.9%) than controls (A.R. 7.2%) (*p*-value = 0.042) (Figure 2A). Similarly, the prevalence of anti-Tg antibodies is significantly higher for PV patients (A.R. 6.8%) as compared with controls (A.R. 0.6%) (*p*-value < 0.001) (Figure 2B).

To establish a potential correlation between anti-thyroid antibodies and disease activity, we divided the patient cohort into active and remittent groups based on consensus guidelines (24). There were no statistically significant differences observed for either anti-TPO (Figure 2C) or anti-Tg antibodies (Figure 2D) between active (*n* = 133) and remittent (*n* = 101) patient samples (all *p*-values > 0.05), indicating that disease activity is not related to the levels of anti-thyroid autoantibodies.

### Female PV Patients Have a Higher Prevalence of Anti-Thyroid Antibodies Than Male PV Patients

**Figure 3:**
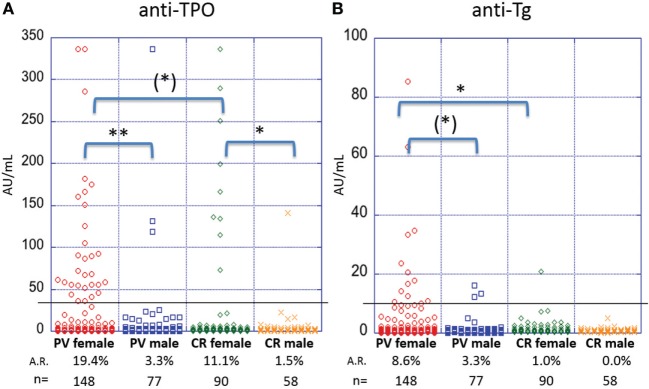
Activity rates of anti-thyroid peroxidase (anti-TPO) and anti-thyroglobulin (anti-Tg) based on sex. Individual dots represent anti-TPO and anti-Tg levels (in AU/ml) in pemphigus vulgaris (PV) patients and controls, both female and male for **(A)** anti-TPO and **(B)** anti-Tg antibodies [(*)*p* < 0.1, *p < 0.05, and **p < 0.01]. Abbreviation: A.R., activity rate. Anti-TPO and anti-Tg antibody rates were modeled as a function of sex using a generalized estimating equations logistic regression model.

A female predominance has been reported for AITD (28). To explore potential differences in autoantibody production between genders, we analyzed serum samples from 148 female and 77 male patients. Female PV patients were found to be significantly more likely to carry anti-TPO antibodies than male patients (A.R. 19.4 vs. 3.3%, respectively, *p*-value > 0.001) (Figure 3A). A similar trend is seen for anti-Tg antibodies (A.R. 8.6% in females vs. 3.3% in males) where the difference in A.R.s trends toward statistical significance (*p* = 0.06) (Figure 3B). Likewise, in the control population, female controls (*n* = 90, 11.1%) were significantly more likely to carry anti-TPO than male controls (*n* = 58, 1.5%) (*p*-value = 0.019) (Figure 3A). However, the A.R. for anti-Tg in females (1.0%) does not differ significantly (*p*-value = 1.00) from that of males (0.0%) (Figure 3B). A significant (or trending toward significant) difference remains between female PV patients and female controls for anti-Tg and anti-TPO, respectively. Such a difference was not observed comparing male patients vs. male controls, potentially due to lower numbers of male samples.

### PV Patients With Cutaneous Only Lesions Are More Likely to Display Anti-Thyroid Antibodies Compared With Other Morphological Subtypes

**Figure 4:**
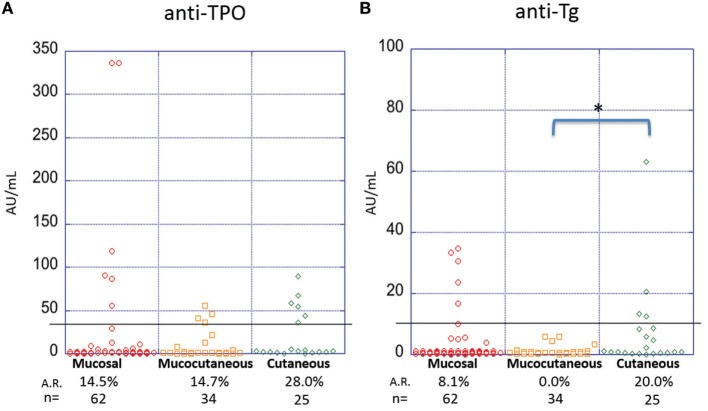
Activity rates of anti-thyroid peroxidase (anti-TPO) and anti-thyroglobulin (anti-Tg) based on morphology. Individual dots represent anti-TPO and anti-Tg levels (in AU/ml) in pemphigus vulgaris patients subgrouped on the basis of lesion morphology for **(A)** anti-TPO and **(B)** anti-Tg antibodies (*p < 0.05). Abbreviation: A.R., activity rate. Anti-TPO and anti-Tg antibody rates were modeled as a function of disease morphology using a generalized estimating equations logistic regression model.

It has been suggested that disease morphology in PV patients is dictated by their anti-desmoglein antibody profile. The desmoglein-compensation hypothesis states that anti-Dsg3 antibodies are sufficient to induce mucosal lesions, while additional anti-Dsg1 antibodies are needed to induce cutaneous lesions (18, 29). To explore a possible relationship between lesion morphology and anti-thyroid antibodies, we compared anti-TPO and anti-Tg levels in PV patients with clearly documented lesional morphology classified into three subtypes: mucosal only, mucocutaneous, and cutaneous only. The prevalence of anti-TPO antibodies in patients with cutaneous only lesions (*n* = 25, A.R. 28.0%) is almost twice that observed in patients with mucosal only (*n* = 62, A.R. 14.5%) or mucocutaneous lesions (*n* = 34, A.R. 14.7%), although this difference does not reach statistical significance (*p*-value 0.409) (Figure 4A). On the other hand, a significant association is observed between anti-Tg antibody A.R. and morphology, where patients with cutaneous only lesions have significantly higher prevalence rates (A.R. 20%) than those with mucosal or mucocutaneous lesions (A.R. 8.1 and 0.0%, respectively) (*p*-value = 0.022) (Figure 4B).

### “HLA-Negative” PV Patients Show a Higher Prevalence of Anti-Thyroid Antibodies Than “HLA-Positive” Patients

**Figure 5:**
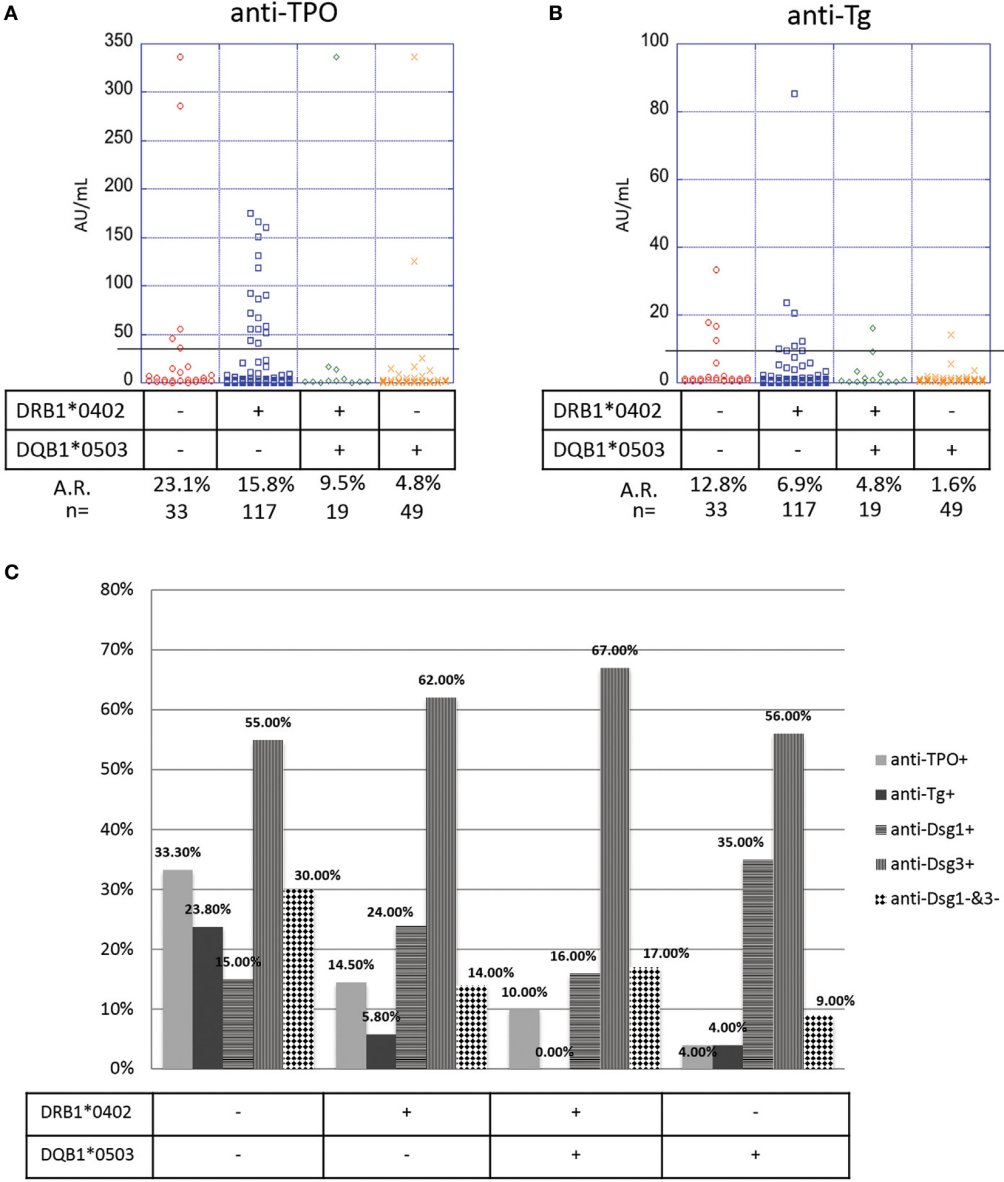
Correlation of anti-thyroid peroxidase (anti-TPO), anti-thyroglobulin (anti-Tg), anti-Dsg3, and anti-Dsg1 levels and human leukocyte antigen (HLA) status. Levels of **(A)** anti-TPO and **(B)** anti-Tg antibodies in pemphigus vulgaris (PV) patients, subgrouped on the basis of presence or absence of PV-associated HLA susceptibility alleles. Individual dots represent anti-TPO and anti-Tg levels (in AU/ml). Anti-TPO and anti-Tg antibody rates were modeled as a function of absence or presence of the PV-associated HLA alleles DRB1*0402 and/or DQB1*0503 using a generalized estimating equations logistic regression model. **(C)** Percent positivity of anti-Dsg1, anti-Dsg3, anti-TPO, and anti-Tg in active PV patients, subgrouped based on the presence or absence of the two PV-associated HLA alleles DRB1*0402 and DQB1*0503. The association between HLA and Dsg status was evaluated using Fisher’s exact test.

Many genetic studies in PV have confirmed the presence and etiopathogenetic role of two HLA alleles: DRB1*0402 and DQB1*0503 (13–15). To explore the extent to which HLA affects autoantibody production in PV patients, we subdivided our study population on the basis of presence or absence of one or both of the two PV-susceptibility alleles. We find that in the PV cohort there is a significant association between HLA type and anti-TPO antibody levels (*p*-value = 0.05): DRB1*0402^−^/DQB1*0503^−^ (HLA-negative) patients (*n* = 33) have the highest prevalence of anti-TPO (A.R. 23.1%) followed by DRB1*0402^+^/DQB1*0503^−^ patients (*n* = 117, A.R. 15.8%), DRB1*0402^+^/DQB1*0503^+^ patients (*n* = 19, A.R. 9.5%), and DRB1*0402^−^/DQB1*0503^+^ patients (*n* = 49, A.R. 4.8%) (Figure 5A), suggesting that the absence of DQB1*0503, regardless of the presence of DRB1*0402, predisposes patient to the development of anti-TPO antibodies. No significant association is observed for anti-Tg antibody (*p*-value = 0.09); however, a similar trend is observed with DRB1*0402^−^/DQB1*0503^−^ patients having the highest prevalence of anti-Tg (A.R. 12.8%) followed by DRB1*0402^+^/DQB1*0503^−^ (A.R. 6.9%), DRB1*0402^+^/DQB1*0503^+^ (A.R. 4.8%), and DRB1*0402^−^/DQB1*0503^+^ patients (A.R. 1.6%) (Figure 5B).

To further explore the link between HLA haplotype and autoantibody prevalence in active PV patients (*note*: 125 of the 133 active patients in our study had both HLA typing data and anti-desmoglein levels), we visualized the presence or absence of anti-desmoglein and anti-thyroid autoantibodies in PV patients based on their HLA type. As expected, anti-Dsg3 is the predominant autoantibody in PV patients, regardless of their HLA type (Figure 5C). Interestingly, whereas DRB1*0402^−^/DQB1*0503^+^ patients have higher percentage positivity for anti-Dsg1 antibodies when compared with other HLA types, patients who do not express either of the two PV-susceptibility alleles, i.e., DRB1*0402^−^/DQB1*0503^−^ patients, have a higher likelihood of not bearing anti-Dsg3 or anti-Dsg1 antibodies, suggesting that non-desmoglein autoantibodies may fill this gap (Figure 5C). In addition, there is a significant association between HLA and Dsg status (*p* = 0.023), where HLA-double negative samples are much less likely to be Dsg-double positive. These data, taken together with the data on anti-TPO distribution based on HLA type, suggest that while anti-Dsg3 antibodies can be generated by a majority of PV patients regardless of HLA type; anti-TPO, and perhaps anti-Tg antibodies are preferably generated in PV patients that do not carry the classical PV-susceptibility alleles DRB1*0402 and DQB1*0503 and may be more relevant than anti-Dsg1 antibodies in this subpopulation.

### Anti-Dsg1 and Anti-Dsg3 Negative Active PV Patients Have Higher Anti-Thyroid Activity

**Figure 6:**
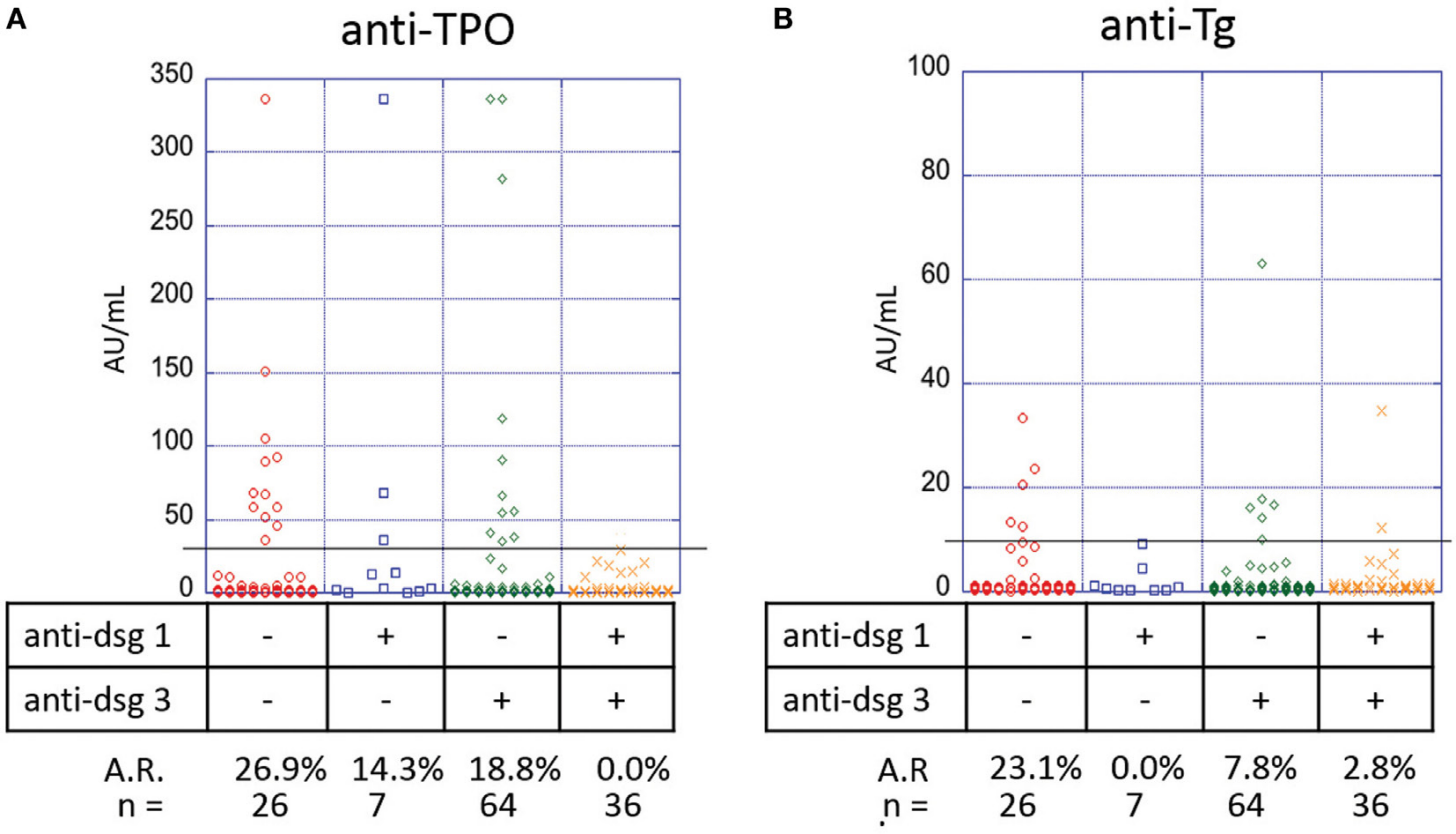
Correlation of anti-thyroid peroxidase (anti-TPO), anti-thyroglobulin (anti-Tg), with anti-Dsg3/1 levels in pemphigus vulgaris (PV) patients with active lesions. Levels of **(A)** anti-TPO and **(B)** anti-Tg antibodies in PV patients, subgrouped on the basis of presence or absence of anti-Dsg3 and or anti-Dsg1 antibodies. Individual dots represent anti-TPO and anti-Tg levels (in AU/ml). Anti-TPO and anti-Tg antibody rates were modeled as a function of absence or presence of anti-Dsg3/1 serum antibodies using a generalized estimating equations logistic regression model.

The pathogenic role of anti-desmoglein antibodies in PV is well established (17–19). To elucidate a potential association between anti-desmoglein and anti-thyroid autoantibodies, we again analyzed the autoantibody profiles of 133 PV patients with active lesions at the time of blood draw. We observed a significant association between the anti-TPO profiles and the absence or presence of anti-desmoglein antibodies (*p*-value 0.019), where anti-Dsg1^−^/3^−^ patients (*n* = 26) have a higher prevalence of anti-TPO antibodies (26.9%) than anti-Dsg1^−^/3^+^ patients (*n* = 64, 18.8%), anti-Dsg1^+^/3^−^ patients (*n* = 7, 14.3%), and anti-Dsg1^+^/3^+^ patients (*n* = 36, 3.9%) (Figure 6A).

A similar trend is observed for anti-Tg antibody (*p*-value 0.032) with anti-Dsg1^−^/3^−^ patients having the highest prevalence (23.1%) followed by anti-Dsg1^−^/3^+^ patients (7.8%), anti-Dsg1^+^/3^+^ patients (2.8%), and anti-Dsg1^+^/3^−^ patients (0.0%) (Figure 6B), suggesting that the absence of both anti-desmoglein antibodies is correlated with the highest anti-thyroid activity, followed by the absence of anti-Dsg1 alone.

Interestingly, Dsg1^−^/3^−^ PV patients in active disease show significantly higher levels of anti-Tg (5.35 ± 8.71 AU/ml) than Dsg1^−^/3^−^ patients in remission (*n* = 44; 1.58 ± 3.08 AU/ml, *p* = 0.01). While anti-TPO levels were also higher in Dsg1^−^/3^−^ patients in active disease (30.71 ± 74.18 AU/ml) than remission (16.16 ± 34.08 AU/ml), this comparison did not reach significance (*p* = 0.28).

### Different Determinants of Disease Expression, Activity, and Phenotype Tend to Cluster Together

**Figure 7:**
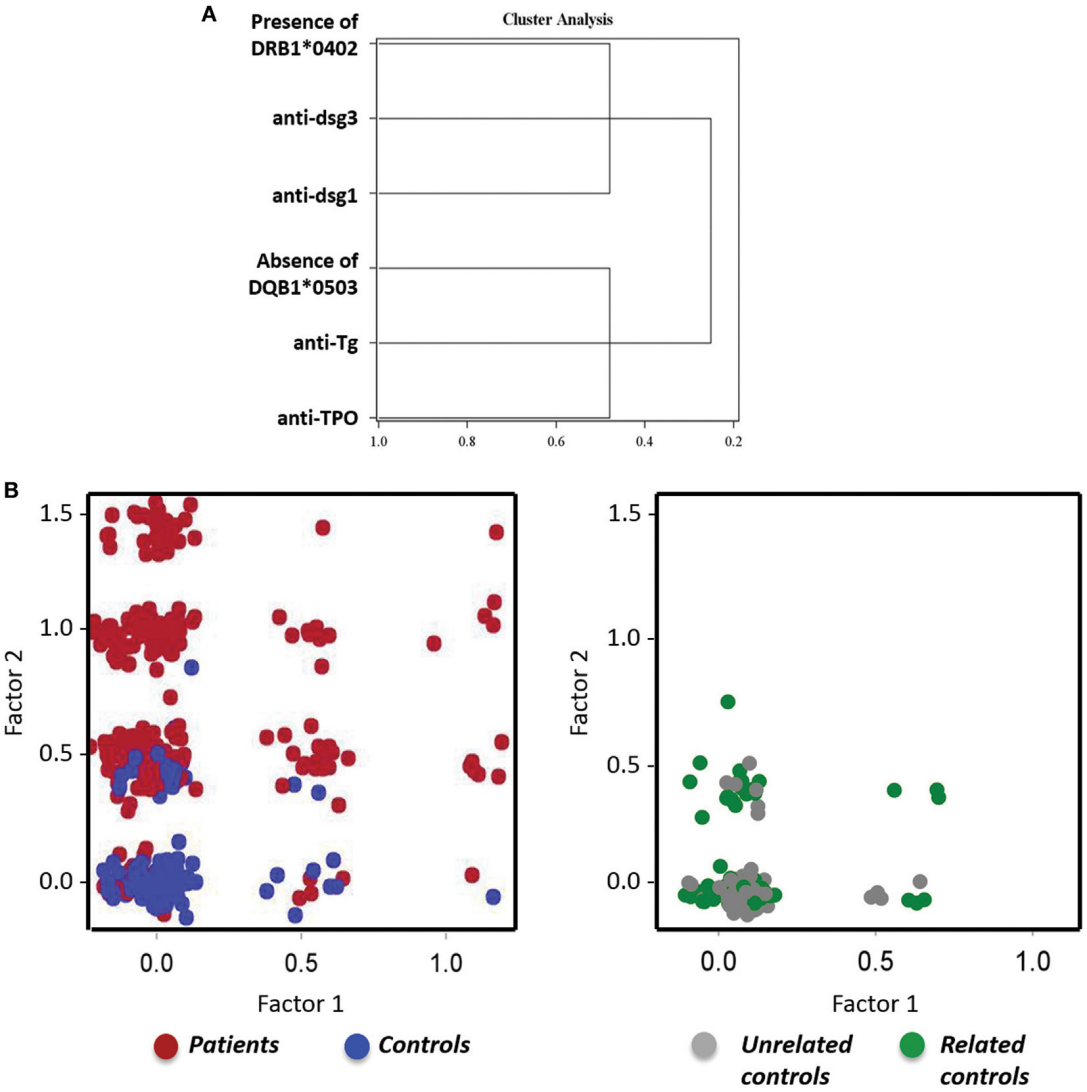
Distinct clustering of anti-Dsg antibodies, anti-thyroid antibodies, and human leukocyte antigen (HLA). **(A)** A tree diagram visually demonstrates the structure and relationship of the clustering variables with anti-Dsg3, anti-Dsg1, and HLA DRB1*0402 in one cluster, and anti-thyroid peroxidase (anti-TPO), anti-thyroglobulin (anti-Tg), and DQB1*0503 a second, distinct cluster. **(B)** Scatter plot representation of pemphigus vulgaris (PV) patients and controls (left) and controls either related or unrelated to PV patients (right), with factor 1 (anti-TPO, anti-TG, and DRB1*0503) on the *x*-axis and factor 2 (anti-Dsg3, anti-Dsg1, and DQB1*0402) on the *y*-axis.

To assess further how and if the variables analyzed in our population relate to each other, principle component analysis was used to identify potential clustering of these variables. The analysis identified two distinct clusters (Figure 7A), which explain 48% of the variation. The first cluster includes presence of anti-TPO and anti-Tg antibodies, in the absence of DQB1*0503; while the second cluster is based on the association of anti-Dsg1, anti-Dsg3, and DRB1*0402 status. All variables show a much stronger positive correlation with their own cluster than the adjacent cluster (Table S1 in Supplementary Material), indicating that while the presence of DRB1*0402 may predispose patients to develop anti-Dsg3 and anti-Dsg1 antibodies, the absence of DQB1*0503 may predispose them to develop anti-TPO and anti-Tg antibodies.

To assess how theses clusters relate to the patient and control populations, factor scores were generated for each cluster using the corresponding standardized scoring coefficients. These factor scores were then plotted against one-another using a scatter plot, with the study sample cohorts represented by different colors (factor 1 = anti-TPO, anti-Tg, and absence of DQB1*0503; factor 2 = anti-Dsg1, anti-Dsg3, and presence of DRB1*0402). The resulting figure shows that PV patients have higher factor scores as compared with controls which tend to cluster toward low factor scores (Figure 7B, left), indicating that one or more variables are present in a given patient within the respective factor groups, but are lacking in the majority of controls. However, a minority of controls are positive for the PV-related factors. To further characterize the control population, we divided healthy controls into PV-related and PV-unrelated individuals. We found that related controls (first-, second-, or third-degree relation to a PV patient) have higher factor scores than unrelated controls (Figure 7B, right), which is particularly true for factor 2 that comprises of anti-Dsg1, anti-Dsg3 antibodies and presence of DRB1*0402. This finding is in line with previous work from our lab that found increased autoantibody levels, including those for anti-Dsg3 and anti-TPO, in PV patients and their relatives by multiplexed autoantigen array when compared with unrelated healthy controls (22).

## Discussion

Autoimmune thyroid disease is among the most commonly diagnosed autoimmune diseases in the general population. Various epidemiological studies have shown the prevalence of AITD to be 7–8% (28, 30, 31), with a reported female predominance of 9:1. The clinical manifestations of AITD may vary from hypothyroidism (Hashimoto’s thyroiditis) to hyperthyroidism (Grave’s disease). The diagnosis of AITD is made based on clinical findings, thyroid function abnormalities as well as the presence of either one of the anti-thyroid antibodies; anti-TPO, anti-Tg or anti-thyroid-stimulating hormone receptor antibodies.

Multiple previous studies from our group and others have reported a higher prevalence of AITD in patients with PV (4, 6–10). In fact, AITD is the most commonly self-reported autoimmune disease in PV patients and/or their first-degree relatives (4, 8). Similarly, levels of AITD-related antibodies, particularly anti-TPO, have been reported to be significantly elevated in PV patients when compared with controls in ethnically diverse populations in Argentina (9), Iran (3, 23), and Turkey (6), with anti-TPO levels ranging from 16 to 40% in the patient population compared with only 6–12% in the healthy control population. Interestingly, numerous studies found that despite the presence of thyroid disease related autoantibodies, many patients were euthyroid clinically (3, 9). In our North American study population, 10.2% of patients reported a history of AITD compared with only 5.4% in the control population (data not shown; noteworthy, of the healthy controls with a personal history of AITD, all had a family history of autoimmune bullous disease, mainly PV), but a higher number of patients had serum reactivity to autoantibodies classically related to AITD (13.9% anti-TPO and 6.8% anti-Tg reactivity). As in previous studies, the self-reported history of AITD and the objectively determined anti-TPO/anti-Tg levels are not tightly correlated. In fact, only 52% of patients with a positive history of thyroid disease have detectable anti-TPO/anti-Tg levels. Conversely, 13% of patients without a personal or family history of AITD still carried detectable levels of anti-TPO/Tg, suggesting that these patients either have not been diagnosed with AITD, that these autoantibodies are non-pathogenic, or that they may be involved in processes unrelated to thyroid pathology. While the data on autoimmune thyroid conditions in PV patients in our study was self-reported and could not be independently verified by other imaging or clinical data, the rates of anti-TPO and anti-Tg levels from PV patients with a personal history of AITD are substantially higher compared with those with only a family history and those with no history (self or family). In future studies, it will be important to document objective measures of thyroid function such as thyroid-stimulating hormone, thyroxine (T4), and triiodothyronine (T3) serum levels to assess the true overlap of thyroid disease and anti-thyroid autoantibody positivity in PV patients.

The overwhelming majority of patients with PV carry anti-desmoglein 3, and to a lesser extent, anti-Dsg1 antibodies; these antibodies have been shown to be sufficient to induce cell dissociation (*in vitro* and *in vivo*) (32). However, a subgroup of patients does develop lesions in the absence of anti-dsg antibodies [personal observation and Ref. (31)]. To date, it is unclear if autoantibodies with non-dsg specificities contribute to blister formation. However, ongoing work in our lab suggests this may be the case for anti-thyroid peroxidase (TPO) antibodies. Using high-throughput protein microarray technology, we found significant IgG reactivity in PV patients toward TPO, several muscarinic acetylcholine receptor subtypes, as well as the established PV-associated antigen desmoglein 3 (22). Furthermore, we observed that patient derived anti-TPO, similar to anti-desmoglein antibodies or patient derived PVIgG, can (i) activate p38MAPK, (ii) increase intracellular calcium and (iii) induce fragmentation of a keratinocyte monolayer (Sajda et al., manuscript in preparation). Likewise, the depletion of anti-TPO autoAbs inhibits the ability of patient purified IgG (PVIgG) to activate these pathways, suggesting a pathogenic role of anti-TPO antibodies in Pemphigus. In this context, it is interesting that anti-TPO and anti-Tg show the highest prevalence in patients that are anti-Dsg3 and anti-Dsg1 negative (A.R. 26.9 and 23.1%, respectively) and are negative or barely detectable in patients that are double-positive for anti-Dsg3 and anti-Dsg1, suggesting that anti-TPO antibodies may have a compensatory or additive function in the absence of the classical PV-related autoantobodies. Interestingly, we also observe a trend toward a higher prevalence of anti-TPO and anti-Tg antibodies in PV patients with the rare “cutaneous only” phenotype and a significant increase in anti-Tg in anti-Dsg1/3 double-negative patients in active disease vs. remission, suggesting that the “supporting role” anti-thyroid antibodies may play in disease pathogenesis is more pronounced in less common disease phenotypes.

A role for anti-TPO antibodies has been previously suggested in autoimmune conditions of non-thyroid origin other than PV, including type I diabetes mellitus (33, 34) and RA (35). Interestingly, we have found both DM type I and RA to be frequent autoimmune comorbidities in PV (4, 10). In fact, these diseases belong to a distinct autoimmune disease cluster along with PV and AITD (8), suggesting common genetic elements across clinically distinct diseases that might underlie autoimmune susceptibility. Autoimmune diseases are multifactorial in origin, with susceptibility controlled by genetic and environmental factors. Thus far, the strongest genetic associations for a wide range of autoimmune conditions, including PV, AITD, RA, and DM type 1, have been with variants in the HLA region (36). PV has a particularly strong associations with HLA with ~ 95% of North American patients carrying one of two HLA class II susceptibility alleles, DRB1*0402 and/or DQB1*0503 (13, 14, 37). Wucherpfennig et al. showed that autoaggressive T cells recognize a limited set of Dsg3 peptides presented by DRB1*0402, thus providing a compelling explanation for the observed association of disease expression and HLA haplotype in PV (16). To date, no such clear association has been found for DQB1*0503. Equally, it is not clear if and how other HLA alleles (non-DRB1*0402 or non-DQB1*0503) link with disease-relevant autoantibodies.

Our data indicate that patients who do not carry the prevalent PV-susceptibility HLA alleles DRB1*0402 and DQB1*0503 are more likely to have higher levels of anti-thyroid antibodies. Interestingly, many of these patients carry alleles in the HLA region that have been associated with thyroid autoimmunity (38, 39), such as DRB1*04, DQB1*0302, DQB1*0301 and DQA*0301 (40) (*data not shown*). Of note, HLA DRB1*0402 has been suggested to be in linkage disequilibrium with DQB1*0302, an allele that has been suggested as a disease causing allele for AITD (41), which may be one of the reasons for the significant overrepresentation of AITD-related autoantibodies in PV. Interestingly, current literature also suggests that DQB1*05 alleles are protective against development of AITD in pediatric DM type I patients (41), and, thus, it is not entirely surprising that we see the lowest levels of anti-TPO and anti-Tg antibodies in the presence of DQB1*0503 allele. Indeed, our cluster analysis reveals that the presence of DRB1*0402 may predispose patients to develop anti-Dsg3 and anti-Dsg1 antibodies, while the absence of DQB1*0503 may predispose them to develop anti-TPO and anti-Tg antibodies.

We did observe that female PV patients have higher antibody A.R.s than either male PV patients or controls. The discrepancy between female and male patients can likely be explained by higher prevalence of AITD in females in general, since there is no large difference in the relative female predominance in either PV patient or controls in our study. However, the exact mechanism behind the increased susceptibility of females to several autoimmune diseases, including pemphigus and AITD, remains to be established.

Taken together, our data support a role for non-desmoglein autoantibodies in PV, specifically anti-TPO antibodies. Our findings further suggest that anti-TPO reactivity in PV is driven by genetic markers that may be in linkage disequilibrium with the established PV-susceptibility alleles and that this association drives the selection of a combination of anti-Dsg and anti-TPO antibodies, with anti-TPO filling the gap in active patients that do not carry the established PV-associated autoantibodies and/or are lacking the established PV-HLA-susceptibility alleles. Understanding the genetic underpinnings and potential mechanistic interplay of anti-desmoglein and non-desmoglein autoantibodies relevant to disease pathogenesis and expression is crucial to improve both treatment and clinical decision making in autoimmune blistering conditions. Further studies are needed to determine both the potential functional correlates of anti-TPO and anti-Tg autoantibodies in the context of cell dissociation and whether the anti-TPO/anti-Tg antibodies detected in PV are similar or identical in specificity and affinity to those detected AITD.

## Ethics Statement

This study was carried out in accordance with the recommendations of the Institutional Review Boards at Weill Cornell Medical College, Michigan State University, and the University at Buffalo with written informed consent from all subjects. All subjects gave written informed consent in accordance with the Declaration of Helsinki. The protocol was approved by the review boards at Weill Cornell Medical College, Michigan State University and the University at Buffalo. No vulnerable populations were involved in this study.

## Author Contributions

KS-S and AS designed the study and enrolled the patients included in this study. KS-S and SK performed the experiments and analyzed the data. KS-S, SK, and AS wrote the manuscript. KA performed the statistical analysis of the final data set. JG provided high resolution HLA typing.

## Conflict of Interest Statement

The authors declare that the research was conducted in the absence of any commercial or financial relationships that could be construed as a potential conflict of interest. The reviewer MK and handling Editor declared their shared affiliation.
