# Supplementary material for: Anti-Thyroid Peroxidase Reactivity Is Heightened in Pemphigus Vulgaris and Is Driven by Human Leukocyte Antigen Status and the Absence of Desmoglein Reactivity
Source: Front Immunol. 2018 Apr 5;9:625. doi: 10.3389/fimmu.2018.00625 (PMC5896579; doi:10.3389/fimmu.2018.00625)
Supplement: Supplementary file 1 [file Table_1.PDF]

Cluster	Variable	R^2 value with own cluster	R^2 value with next cluster
**Cluster 1**	Anti-TPO (+/-)	0.6829	0.0008
	Anti-Tg (+/-)	0.6912	0.0013
	DQB1*0503	0.0448	0.0015
**Cluster 2**	Anti-Dsg1 (+/-)	0.3527	0.0043
	Anti-Dsg3 (+/-)	0.6616	0.0001
	DRB1*0402 (+/-)	0.4473	0.0195

Supplemental Table S1. Correlation of variables with their own cluster vs. the next cluster. The higher the R2 value, the better the correlation among the cluster variables.
